# Supplementary material for: Third harmonic characterization of antiferromagnetic heterostructures
Source: Nat Commun. 2022 Jun 27;13:3659. doi: 10.1038/s41467-022-31451-9 (PMC9237044; doi:10.1038/s41467-022-31451-9)
Supplement: Supplementary file 1 — Supplementary Information [file 41467_2022_31451_MOESM1_ESM.pdf]

Supplementary Information for
Third Harmonic Characterization of Antiferromagnetic Heterostructures

Yang Cheng,[1] Egecan Cogulu,[2] Rachel D. Resnick,[1] Justin J. Michel,[1] Nahuel N. Statuto,[2] Andrew D. Kent,[2] and Fengyuan Yang[1]

[1]Department of Physics, The Ohio State University, Columbus, OH 43210, USA

[2]Department of Physics, Center for Quantum Phenomena, New York University, New York, NY 10003, USA

1) X-ray diffraction (XRD) of α-Fe_2O_3/Al_2O_3(0001) film

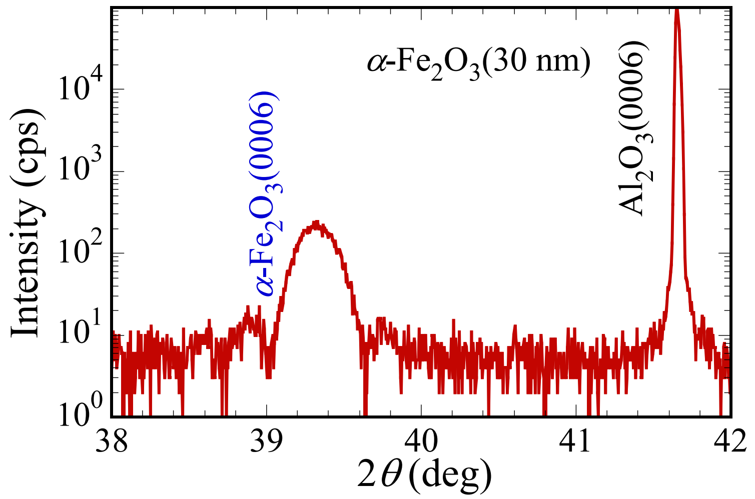

Figure S1. XRD scan of a 30 nm α-Fe_2O_3 epitaxial film on Al_2O_3(0001) with clear Laue oscillations, demonstrating the high uniformity of the α-Fe_2O_3 film.

2) In-plane angular-dependent Harmonic voltages in Pt/α-Fe_2O_3 (0001)

When an AC current $I = I_0 \sin(\omega t)$ is applied, due to spin-orbit torque (SOT) and thermoelectric effects, the measured transverse voltage $V = \sum_n V_{n\omega} \sin(n\omega t + \phi)$ where $V_{n\omega}$ is called n^{th} harmonic voltage. Here we show the harmonic voltages in Pt/α-Fe_2O_3 (0001) in an in-plane magnetic field.

Current-induced spin-orbit torques and magnetoelastic effect drive the antiferromagnetic (AFM) moment slightly off the equilibrium orientation, which change the transverse resistance of

Pt/α-Fe_2O_3 (0001) bilayers. Thus,

$$V = IR(I) = I\left(R_0 + I\frac{dR}{dI}|_{I=0} + \frac{1}{2}I^2\frac{d^2R}{dI^2}|_{I=0}\right)$$

$$= V_0 + V_{1\omega}\sin(\omega t) + V_{2\omega}\cos(2\omega t) + V_{3\omega}\sin(3\omega t), \quad \text{(S1-1)}$$

where R_0 is the equilibrium transverse resistance at the limit of $I = 0$, and,

$$V_0 = \frac{1}{2}{I_0}^2\frac{dR}{dI}|_{I=0}, \quad \text{(S1-2)}$$

$$V_{1\omega} = I_0R_0 + \frac{3}{8}{I_0}^3\frac{d^2R}{dI^2}|_{I=0} \approx I_0R_0, \quad \text{(S1-3)}$$

$$V_{2\omega} = -\frac{1}{2}{I_0}^2\frac{dR}{dI}|_{I=0}, \quad \text{(S1-4)}$$

$$V_{3\omega} = -\frac{1}{8}{I_0}^3\frac{d^2R}{dI^2}|_{I=0}, \quad \text{(S1-5)}$$

where $V_{1\omega}$, $V_{2\omega}$ and $V_{3\omega}$ are the first, second, and third harmonic voltages, which are proportional to I_0, ${I_0}^2$ and ${I_0}^3$, respectively.

Figure S2 shows the schematics of two sublattice magnetization $\mathbf{m_{A(B)}}$ of α-Fe_2O_3 in an in-plane magnetic field $\mathbf{H}$ (θ_H= 90°) within a spherical coordinate system with polar angle θ and azimuthal angle φ for each vector. We define unit vector of the Néel order $\mathbf{n} = \frac{\mathbf{m_A}-\mathbf{m_B}}{|\mathbf{m_A}-\mathbf{m_B}|}$ and the net magnetization $\mathbf{m} = \mathbf{m_A} + \mathbf{m_B}$.

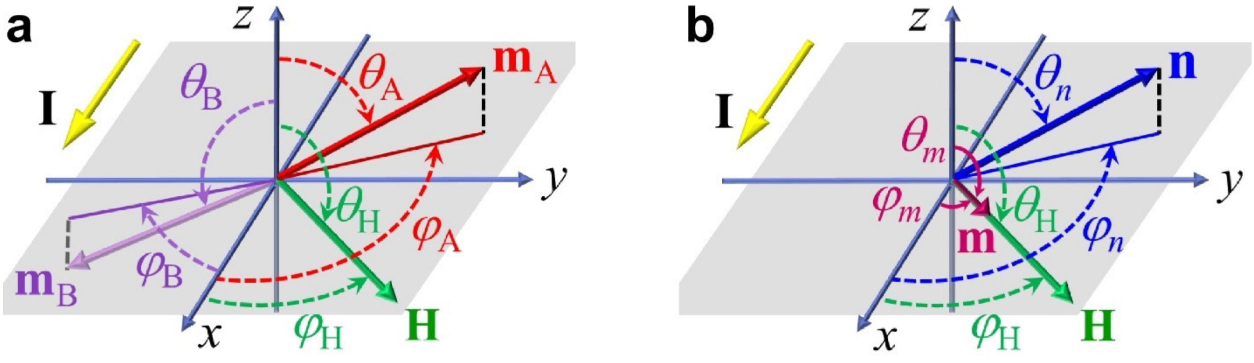

Figure S2. Schematics of **a**, two spin sublattices $\mathbf{m_{A(B)}}$ and **b**, unit vector of Néel order **n** and net magnetization **m** of α-Fe_2O_3 in the presence of an in-plane magnetic field **H** within a spherical coordinate system with polar angle θ and azimuthal angle φ for each of the vectors: $\mathbf{m_A}$ (brown), $\mathbf{m_B}$ (purple), **n** (blue), **m** (red), and **H** (green).

Next, we show the torque balance equations, which are similar to those for ferromagnets,

$$-\mathbf{m_A} \times \mathbf{H_A^{eff}} + \boldsymbol{\tau}_\mathbf{A}^\mathbf{DL} + \boldsymbol{\tau}_\mathbf{A}^\mathbf{FL} = 0, \tag{S2-1}$$

$$-\mathbf{m_B} \times \mathbf{H_B^{eff}} + \boldsymbol{\tau}_\mathbf{B}^\mathbf{DL} + \boldsymbol{\tau}_\mathbf{B}^\mathbf{FL} = 0, \tag{S2-2}$$

where $\mathbf{H_{A(B)}^{eff}} = \mathbf{H} - H_{\mathrm{ex}}\mathbf{m_{B(A)}} + \mathbf{H_{A(B)}^{DM}} + \mathbf{H_{A(B)}^{an}}$ is the effective magnetic field that includes the following contributions:

- External magnetic field $\mathbf{H}$,
- Exchange field H_{ex},
- Effective field of Dzyaloshinskii-Moriya (DM) interaction $\mathbf{H_{A(B)}^{DM}} = \pm H_{\mathrm{DM}}\hat{\mathbf{z}} \times \mathbf{m_{B(A)}}$,
- Anisotropy field $\mathbf{H_{A(B)}^{an}} = -m_{\mathrm{A(B)}}^{z} H_{\mathrm{K}}\hat{\mathbf{z}} + m_{\mathrm{A(B)}}^{x} H_{\mathrm{ME}}\hat{\mathbf{x}}$.[1] ($H_{\mathrm{K}}$ is the easy-plane anisotropy. H_{ME} is the effective easy axis anisotropy due to magnetoelastic (ME) effect).

We assume the external magnetic field is large enough so that the α-Fe_2O_3 film is in single domain state and we ignore the small in-plane tri-axial anisotropy. The field-like torque $\boldsymbol{\tau}_\mathbf{A(B)}^\mathbf{FL}$ and the damping-like torque $\boldsymbol{\tau}_\mathbf{A(B)}^\mathbf{DL}$ are,

$$\boldsymbol{\tau}_\mathbf{A(B)}^\mathbf{FL} = H_{\mathrm{FL}}\hat{\boldsymbol{\sigma}} \times \mathbf{m_{A(B)}}, \tag{S3-1}$$

$$\boldsymbol{\tau}_\mathbf{A(B)}^\mathbf{DL} = H_{\mathrm{DL}}\mathbf{m_{A(B)}} \times (\hat{\boldsymbol{\sigma}} \times \mathbf{m_{A(B)}}), \tag{S3-2}$$

where $\hat{\boldsymbol{\sigma}}$ is the unit vector of spin polarization in Pt along $\hat{\mathbf{y}}$, H_{FL} and H_{DL} are the effective fields of field-like and damping-like torques, respectively. Here $H_{\mathrm{ME}} \propto \nabla T \propto I^2$, $H_{\mathrm{FL}} \propto I$, and $H_{\mathrm{DL}} \propto I$, where ∇T is the current-induced temperature gradient along $\mathbf{z}$.[1]

At $I = 0$ with the external magnetic field applied in the xy plane, the equilibrium orientations of the sublattice $\mathbf{m_A}$ and $\mathbf{m_B}$ as well as the Néel order $\mathbf{n}$ and net magnetization $\mathbf{m}$ can be described by the following polar and azimuthal angles (see Fig. S2),

$$\theta_{\mathrm{A0}} = \theta_{\mathrm{B0}} = \theta_{n0} = \theta_{m0} = \frac{\pi}{2}, \tag{S4-1}$$

$$\varphi_{\mathrm{A0}} = \varphi_{\mathrm{H}} + \frac{\pi}{2} - \frac{H+H_{\mathrm{DM}}}{2H_{\mathrm{ex}}}, \tag{S4-2}$$

$$\varphi_{\mathrm{B0}} = \varphi_{\mathrm{H}} - \frac{\pi}{2} + \frac{H+H_{\mathrm{DM}}}{2H_{\mathrm{ex}}}, \tag{S4-3}$$

$$\varphi_{n0} = \varphi_{\mathrm{H}} + \frac{\pi}{2}, \tag{S4-4}$$

$$\varphi_{m0} = \varphi_{\mathrm{H}}. \tag{S4-5}$$

When $I \neq 0$, $\mathbf{m}_{\mathbf{A(B)}}$ deviates from its equilibrium orientation. By separately solving the torque balance equations [Eq. (S2)] with non-zero H_{FL}, $H_{\mathrm{DL}}$and H_{ME} (assuming H_{FL}, H_{DL}, and $H_{\mathrm{ME}} \ll H_{\mathrm{K}} \ll H \ll H_{\mathrm{ex}}$), we can obtain the new orientations of $\mathbf{m}_{\mathbf{A}}$, $\mathbf{m}_{\mathbf{B}}$, $\mathbf{n}$ and $\mathbf{m}$ described by the change of polar (azimuthal) angles $\Delta\theta(\varphi)_{A,B,n,m}$ as shown in Eqs. (S5)-(S7) below.

For field-like torque,

$$\Delta\theta_{\mathrm{A}} = \Delta\theta_{\mathrm{B}} = \Delta\theta_{n} = \Delta\theta_{m} = 0, \tag{S5-1}$$

$$\Delta\varphi_{\mathrm{A}} = \Delta\varphi_{\mathrm{B}} = \Delta\varphi_{n} = \Delta\varphi_{m} = \frac{H_{\mathrm{FL}}}{H}\cos\varphi_{\mathrm{H}}. \tag{S5-2}$$

For damping-like torque,

$$\Delta\theta_{\mathrm{A}} = -\Delta\theta_{\mathrm{B}} = \Delta\theta_{n} = \frac{H_{\mathrm{DL}}}{H_{\mathrm{K}}+H_{\mathrm{DM}}(\frac{H+H_{\mathrm{DM}}}{2H_{\mathrm{ex}}})}\sin\varphi_{\mathrm{H}},\ \Delta\theta_{m} = 0, \tag{S6-1}$$

$$\begin{aligned}\Delta\varphi_{\mathrm{A}} = \Delta\varphi_{\mathrm{B}} = \Delta\varphi_{n} = \Delta\varphi_{m} &= -\frac{2H_{\mathrm{ex}}H_{\mathrm{DL}}}{H(H+H_{\mathrm{DM}})}\cos\varphi_{\mathrm{H}}\Delta\theta_{\mathrm{A}} \\ &= -\frac{H_{\mathrm{ex}}H_{\mathrm{DL}}{}^{2}}{H(H+H_{\mathrm{DM}})(H_{\mathrm{K}}+H_{\mathrm{DM}}(\frac{H+H_{\mathrm{DM}}}{2H_{\mathrm{ex}}}))}\sin 2\varphi_{\mathrm{H}},\end{aligned} \tag{S6-2}$$

As we can see from Eqs. S6-1 and S6-2, $\Delta\theta_{\mathrm{A}} = -\Delta\theta_{\mathrm{B}}$ and $\Delta\varphi_{\mathrm{A}} = \Delta\varphi_{\mathrm{B}}$. This is because the opposite sublattices act symmetrically on H_{DL} which tilts them out-of-plane in opposite directions. In-plane tilting would not occur due to the dominant antiferromagnetic exchange coupling between the sublattices. Mathematically, at equilibrium state, the two sublattice magnetization $\mathbf{m}_{\mathbf{A}}$ and $\mathbf{m}_{\mathbf{B}}$ stay in-plane antiparallel with each other. Under the damping-like torque where the effective field $\mathbf{H}^{\mathbf{DL}}_{\mathbf{A(B)}} \propto \mathbf{m}_{\mathbf{A(B)}} \times \boldsymbol{\sigma}$, $\mathbf{m}_{\mathbf{A}}$ and $\mathbf{m}_{\mathbf{B}}$ tilt towards $\boldsymbol{z}$ with $\Delta m_{\mathrm{A}}{}^{\mathrm{z}} = -\Delta m_{\mathrm{B}}{}^{\mathrm{z}}$. As a result, we have $\Delta\theta_{\mathrm{A}} = -\Delta\theta_{\mathrm{B}}$. Then, the out-of-plane component of sublattice magnetization experiences the other damping-like torque with $\Delta\mathbf{H}^{\mathbf{DL}}_{\mathbf{A(B)}} \propto \Delta\mathbf{m}_{\mathbf{A(B)}} \times \boldsymbol{\sigma}$, which leads to $\Delta\varphi_{\mathrm{A}} = \Delta\varphi_{\mathrm{B}}$. Thus, there is no in-plane tilting. For magnetoelastic effect,

$$\Delta\theta_{\mathrm{A}} = \Delta\theta_{\mathrm{B}} = \Delta\theta_{n} = \Delta\theta_{m} = 0; \tag{S7-1}$$

$$\Delta\varphi_{\mathrm{A}} = \Delta\varphi_{\mathrm{B}} = \Delta\varphi_{n} = \Delta\varphi_{m} = \frac{H_{\mathrm{ex}}H_{\mathrm{ME}}}{H(H+H_{\mathrm{DM}})}\sin 2\varphi_{\mathrm{H}} \tag{S7-2}$$

To check the explicit solution of Eqs. (S5)-(S7), we numerically solve the coupled Landau-Lifshitz-Gilbert (LLG) equations,[2]

$$\frac{d\mathbf{m_A}}{dt} = -\gamma \mathbf{m_A} \times \mathbf{H_A^{eff}} + \gamma \boldsymbol{\tau}_\mathbf{A}^\mathbf{DL} + \gamma \boldsymbol{\tau}_\mathbf{A}^\mathbf{FL} + \alpha \mathbf{m_A} \times \frac{d\mathbf{m_A}}{dt}, \quad \text{(S8-1)}$$

$$\frac{d\mathbf{m_B}}{dt} = -\gamma \mathbf{m_B} \times \mathbf{H_B^{eff}} + \gamma \boldsymbol{\tau}_\mathbf{B}^\mathbf{DL} + \gamma \boldsymbol{\tau}_\mathbf{B}^\mathbf{FL} + \alpha \mathbf{m_B} \times \frac{d\mathbf{m_B}}{dt}, \quad \text{(S8-2)}$$

where γ is gyromagnetic ratio and α is Gilbert damping constant. Figure S3 shows the simulated results, which agree well with the explicit solution using Eqs. (S5)-(S7).

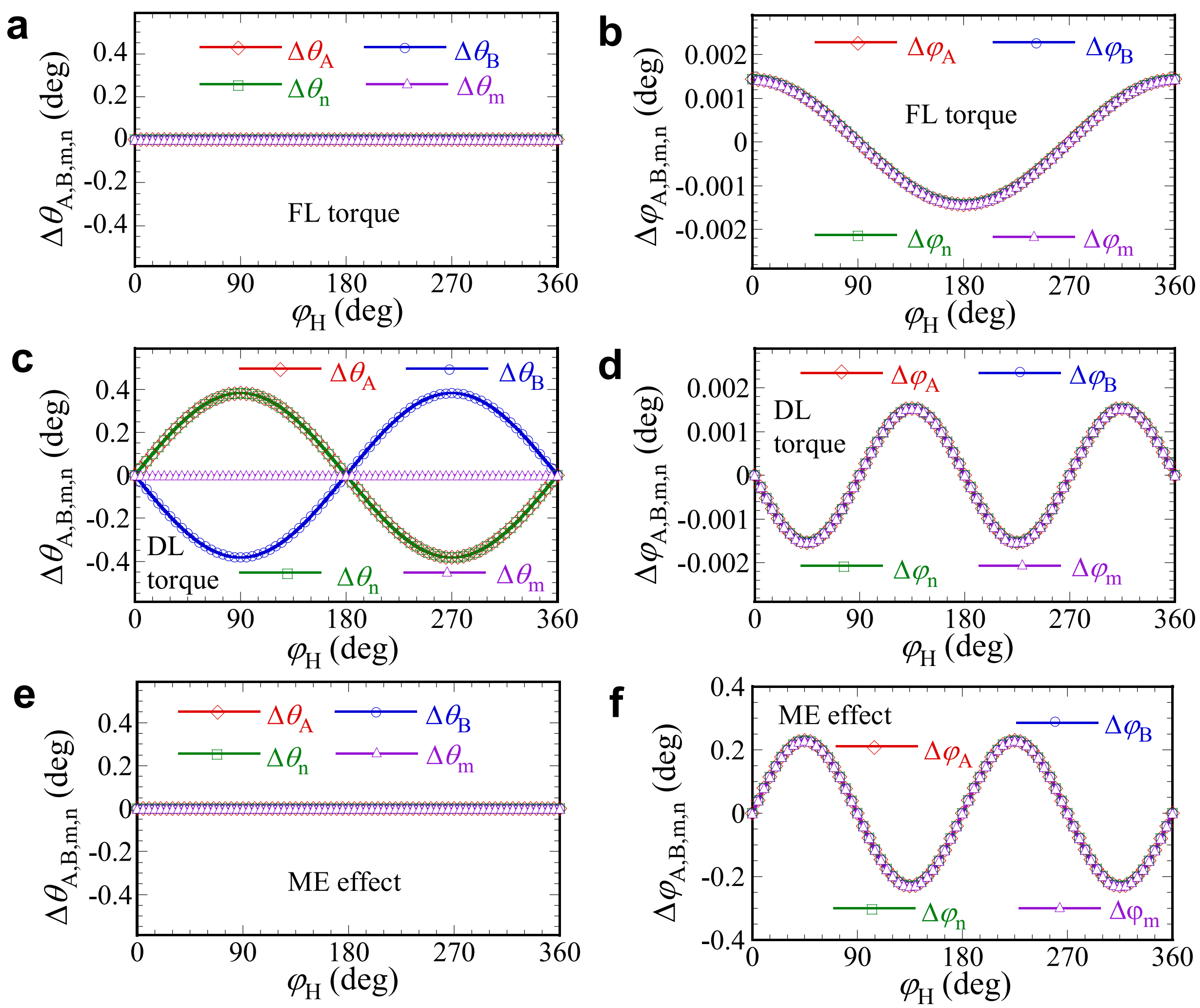

Figure S3. Simulation of $\Delta\theta(\varphi)_{A,B,n,m}$ for a Pt(5 nm)/α-Fe_2O_3(30 nm) bilayer under **a**, **b**, field-like torque, **c**, **d**, damping-like torque, and **e**, **f**, magnetoelastic effect, using $H = 4 \times 10^4$ Oe, $H_{ex} = 9 \times 10^6$ Oe, $H_{DM} = 1.6 \times 10^4$ Oe, $H_K = 100$ Oe, $H_{FL} = H_{DL} = H_{ME} = 1$ Oe. Lines are plotted using explicit solution of Eqs. (S5)-(S7). Symbols are calculated using Eq. (S8).

Using Eqs. (S5)-(S6), we can obtain different angular dependencies of Harmonic voltage components for field-like torque, damping-like torque and magnetoelastic effect. For Pt/α-Fe_2O_3 (0001) in an in-plane magnetic field **H**,

$$R_0 = R_{\mathrm{TSMR}}\sin^2\theta_{n0}\sin2\varphi_{n0} = -R_{\mathrm{TSMR}}\sin 2\varphi_{\mathrm{H}}, \quad \text{(S9)}$$

which is called the negative transverse spin Hall magnetoresistance (TSMR).[3,4] Then, the derivative resistance terms in Eq. (S1) can be derived as,

$$\frac{dR}{dI}|_{I=0} = 2R_{\mathrm{TSMR}}\sin^2\theta_n\cos(2\varphi_n)\frac{d\varphi_n}{dI}|_{I=0}, \quad \text{(S10-1)}$$

$$\frac{d^2R}{dI^2}|_{I=0} = 2R_{\mathrm{TSMR}}\sin^2\theta_n\cos(2\varphi_n)\frac{d^2\varphi_n}{dI^2}|_{I=0} - 4R_{\mathrm{TSMR}}\sin^2\theta_n\sin(2\varphi_n)\left(\frac{d\varphi_n}{dI}|_{I=0}\right)^2$$

$$\approx 2R_{\mathrm{TSMR}}\sin^2\theta_n\cos(2\varphi_n)\frac{d^2\varphi_n}{dI^2}|_{I=0}. \quad \text{(S10-2)}$$

Here we neglect higher order terms. Thus,

$$V_{1\omega} = I_0R_0 = -I_0R_{\mathrm{TSMR}}\sin 2\varphi_{\mathrm{H}} = -V_{\mathrm{TSMR}}\sin 2\varphi_{\mathrm{H}} \quad \text{(S11)}$$

For field-like torque term,

$$V_{2\omega}^{\mathrm{FL}} = -\frac{1}{2}{I_0}^2\frac{dR}{dI}|_{I=0} = V_{\mathrm{TSMR}}\frac{H_{\mathrm{FL}}}{H}\cos(2\varphi_{\mathrm{H}})\cos\varphi_{\mathrm{H}} \quad \text{(S12-1)}$$

$$V_{3\omega}^{\mathrm{FL}} = -\frac{1}{8}{I_0}^3\frac{d^2R}{dI^2}|_{I=0} = 0 \quad \text{(S12-2)}$$

For damping-like torque,

$$V_{2\omega}^{\mathrm{DL}} = -\frac{1}{2}{I_0}^2\frac{dR}{dI}|_{I=0} = 0 \quad \text{(S13-1)}$$

$$V_{3\omega}^{\mathrm{DL}} = -\frac{1}{8}{I_0}^3\frac{d^2R}{dI^2}|_{I=0} = -V_{\mathrm{TSMR}}\frac{H_{\mathrm{ex}}{H_{\mathrm{DL}}}^2}{4H(H+H_{\mathrm{DM}})(H_{\mathrm{K}}+H_{\mathrm{DM}}(\frac{H+H_{\mathrm{DM}}}{2H_{\mathrm{ex}}}))}\sin 4\varphi_{\mathrm{H}} \quad \text{(S13-2)}$$

For magnetoelastic effect,

$$V_{2\omega}^{\mathrm{ME}} = -\frac{1}{2}{I_0}^2\frac{dR}{dI}|_{I=0} = 0 \quad \text{(S14-1)}$$

$$V_{3\omega}^{\mathrm{ME}} = -\frac{1}{8}{I_0}^3\frac{d^2R}{dI^2}|_{I=0} = V_{\mathrm{TSMR}}\frac{H_{\mathrm{ex}}H_{\mathrm{ME}}}{4H(H+H_{\mathrm{DM}})}\sin 4\varphi_{\mathrm{H}} \quad \text{(S14-2)}$$

We notice that for field-like torque, $\Delta\varphi_n^{FL} \propto H_{\mathrm{FL}} \propto I$, but for damping-like torque and magnetoelastic effect, $\Delta\varphi_n^{\mathrm{DL}} \propto H_{\mathrm{DL}}{}^2 \propto I^2$ and $\Delta\varphi_n^{\mathrm{ME}} \propto H_{\mathrm{ME}} \propto I^2$. Thus, field-like torque only contributes to the second harmonic voltage while damping-like torque and magnetoelastic effect contributes to third harmonic voltage.

In addition, we need to take spin Seebeck effect (SSE) into consideration. Joule heating by applied current generates a temperature gradient $\nabla T \propto I^2$. Since SSE voltage $V_{\mathrm{SSE}} \propto \nabla T$, it will directly contribute to the second harmonic voltage. Similar to ferromagnets, SSE in antiferromagnets is originated from the tilted net magnetization which is in parallel to the external field.[5] Based on the SSE theory, $V_{2\omega}^{\mathrm{SSE}} \propto \nabla T \times \mathbf{m}$, where $\mathbf{m}$ is the net magnetization,[6,7]

$$V_{2\omega}^{\mathrm{SSE}} = V_{\mathrm{SSE}} \cos\varphi_{\mathrm{H}} \tag{S15}$$

In our previous work as well as some other reports,[1,8-11] the applied current directly changes the resistivity of Pt by thermal heating. Besides, heating induced soften of magnetization could also modify the measured TSMR (See Section (6) for more details). In both cases, the change of the resistance $\Delta\mathrm{R} \propto I^2$ contributes to the third harmonic voltage,

$$V_{3\omega}^{\Delta\mathrm{R}} = -\frac{1}{8} I_0{}^3 \frac{d^2R}{dI^2}\Big|_{I=0} = \frac{1}{8} I_0 \Delta\mathrm{R} \sin 2\varphi_{\mathrm{H}} = \frac{1}{8} \Delta V_{\mathrm{TSMR}} \sin 2\varphi_{\mathrm{H}} \tag{S16}$$

Finally, we have all the contributions to the second and third harmonic voltages,

$$V_{2\omega} = V_{2\omega}^{\mathrm{FL}} + V_{2\omega}^{\mathrm{SSE}} = V_{\mathrm{TSMR}} \frac{H_{\mathrm{FL}}}{H} \cos(2\varphi_{\mathrm{H}}) \cos\varphi_{\mathrm{H}} + V_{\mathrm{SSE}} \cos\varphi_{\mathrm{H}} \tag{S17}$$

$$V_{3\omega} = V_{3\omega}^{\mathrm{DL}} + V_{3\omega}^{\mathrm{ME}} + V_{3\omega}^{\Delta\mathrm{R}}$$

$$= V_{\mathrm{TSMR}}\Big(-\frac{H_{\mathrm{ex}} H_{\mathrm{DL}}{}^2}{4H(H+H_{\mathrm{DM}})(H_{\mathrm{K}}+H_{\mathrm{DM}}(\frac{H+H_{\mathrm{DM}}}{2H_{\mathrm{ex}}}))} + \frac{H_{\mathrm{ex}} H_{\mathrm{ME}}}{4H(H+H_{\mathrm{DM}})}\Big) \sin 4\varphi_{\mathrm{H}} + \frac{1}{8} \Delta V_{\mathrm{TSMR}} \sin 2\varphi_{\mathrm{H}} \tag{S18}$$

3) Magnetoelastic effect at different temperatures

As temperature varies, several important parameters such as thermal conductivity, heat capacity and thermal expansion coefficient change considerably, which may impact the Néel order of the antiferromagnets. Table S1 lists all key parameters that are related to magnetoelastic effect. Using COMSOL, we simulate the current-induced anisotropic compressive stress Δσ at different temperatures (see Fig. S4). The size of Al_2O_3 substrate is 5 mm × 5 mm × 0.5 mm, and the Hall bar channel width is 5 μm. The thicknesses of Pt and α-Fe_2O_3 are 5 and 30 nm, respectively, the same as in real samples. From the simulation, Δσ at the center of Hall bar decreases from -

3.73 Mpa at 300 K to -0.67 Mpa at 200 K and -0.02 Mpa at 100 K, and $\Delta\sigma \propto \nabla T$. Since $H_{\mathrm{ME}} \propto \Delta\sigma$, we can use it to calculate $V_{3\omega}^{\mathrm{ME}}$ at different temperatures.

	Pt (100 K)	Pt (200 K)	Pt (300 K)	Al_2O_3 (100 K)	Al_2O_3 (200 K)	Al_2O_3 (300 K)	α-Fe_2O_3 (100 K)	α-Fe_2O_3 (200 K)	α-Fe_2O_3 (300 K)
Young's modulus (Gpa)	164.6	161.2	157.9	368.6	365.7	361.1	228.4	224.2	220.0
Poisson's ratio	0.385	0.385	0.385	0.29	0.29	0.29	0.29	0.29	0.29
Electrical conductivity (S/m)	7.72×10^6	6.39×10^6	5.55×10^6						
Coefficient of thermal expansion (1/K)	6.72×10^{-6}	8.42×10^{-6}	8.94×10^{-6}	6.70×10^{-7}	3.16×10^{-6}	5.10×10^{-6}	2.62×10^{-6}	1.10×10^{-5}	1.70×10^{-5}
Thermal conductivity (W/(m·K))	77.5	72.6	71.6	450	82	27	54	27	18
Density (Kg/m^3)	21480	21465	21450	3990	3989	3987	5258	5257	5250
Heat capacity at constant pressure C_p (J/(Kg·K))	100.2	125.1	133.0	125.9	501.7	778.6	197.9	478.4	651.4

Table S1. Parameters used in the simulation of magnetoelastic effect in a Pt(5 nm)/α-Fe_2O_3(30 nm) bilayer.[12-26] The conductivity of Pt is measured in our sample.

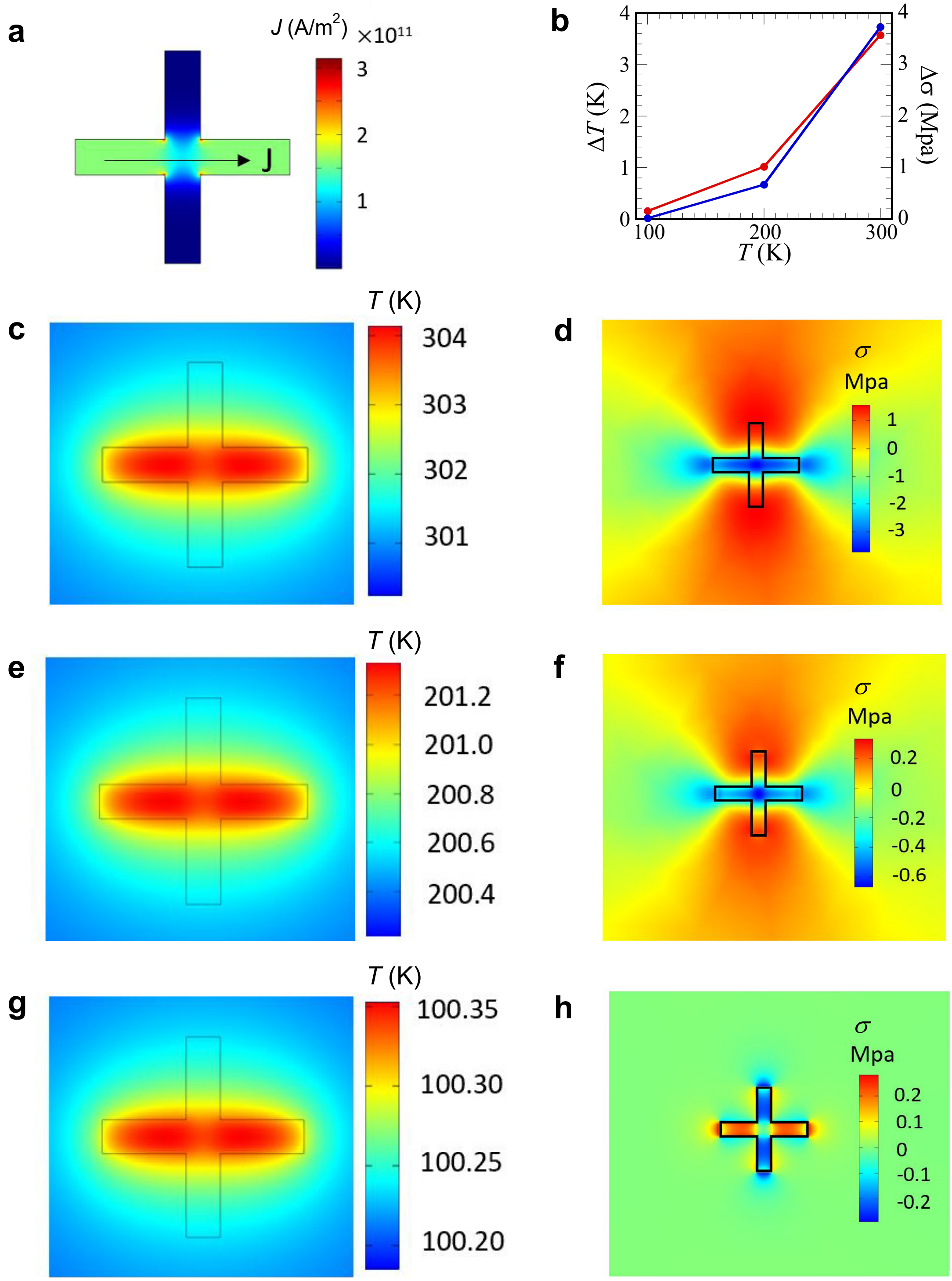

Figure S4. **Current-induced stress in a Pt(5 nm)/*α*-Fe_2O_3(30 nm) bilayer at different temperatures.** **a**, Distribution of current density in the hall bar with I = 4 mA which corresponds to a current density of 1.6×10^{11} $\mathrm{A/m^2}$. **b**, Temperature change (between the center of the sample and the environment) and induced stress at the center of the Hall bar at different temperatures. Color plots of **c**,**e**,**g**, temperature and **d**,**f**,**h**, induced stress at **c**,**d**, T = 300 K, **e**,**f**, T = 200 K, and **g**,**h**, T = 100 K.

4) More harmonic measurement results

Figure S5 shows the different harmonic voltage components at different temperatures. We find that the spin Seebeck coefficient and the effective field that is attributed to field-like torque at multiple domain state have a weak temperature dependence. The conventional field-like torque effective field has a strong temperature dependence as reported in previous research;[27-29] however, the detailed mechanism is under debate, and future study is needed to reveal the underlying physics. The $V_{3\omega}^{\Delta R}$ contribution related to the change of Pt resistivity also decreases with temperature, which is due to the decrease of ΔT as shown in Fig. S4b.

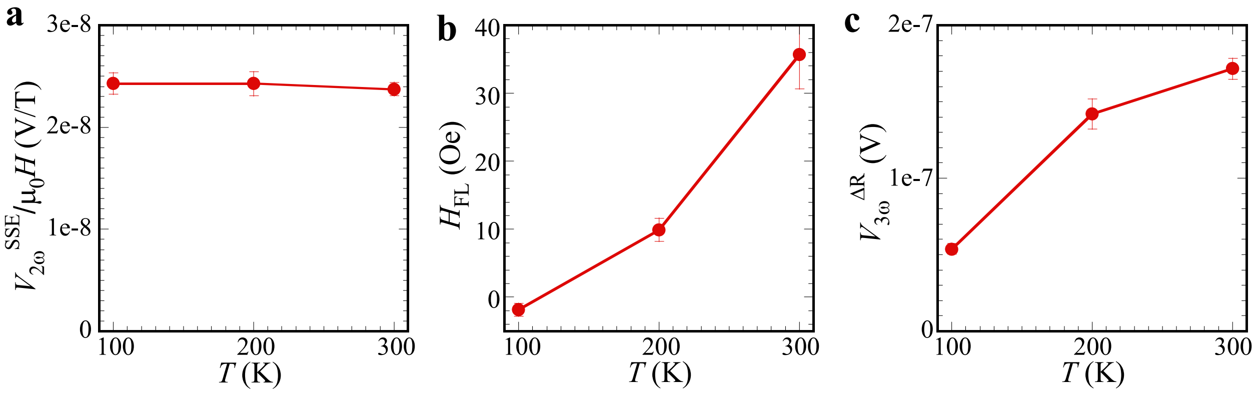

Figure S5. Harmonic voltage components for a Pt(5 nm)/α-Fe_2O_3(30 nm) bilayer at different temperatures. a, Spin Seebeck effect coefficient. **b**, Effective field of field-like torque H_{FL}. **c**, Third harmonic voltage due to resistance change $V_{3\omega}^{\Delta R}$. Error bars represent fitting uncertainty.

We also make similar harmonic measurements for the samples with different α-Fe_2O_3 thicknesses. Figure S6 shows the results of Pt(5 nm)/α-Fe_2O_3 bilayers with the thicknesses of α-Fe_2O_3 from 30 to 10 nm. The 20 and 10 nm samples show similar harmonic voltage components while the magnitude is smaller.

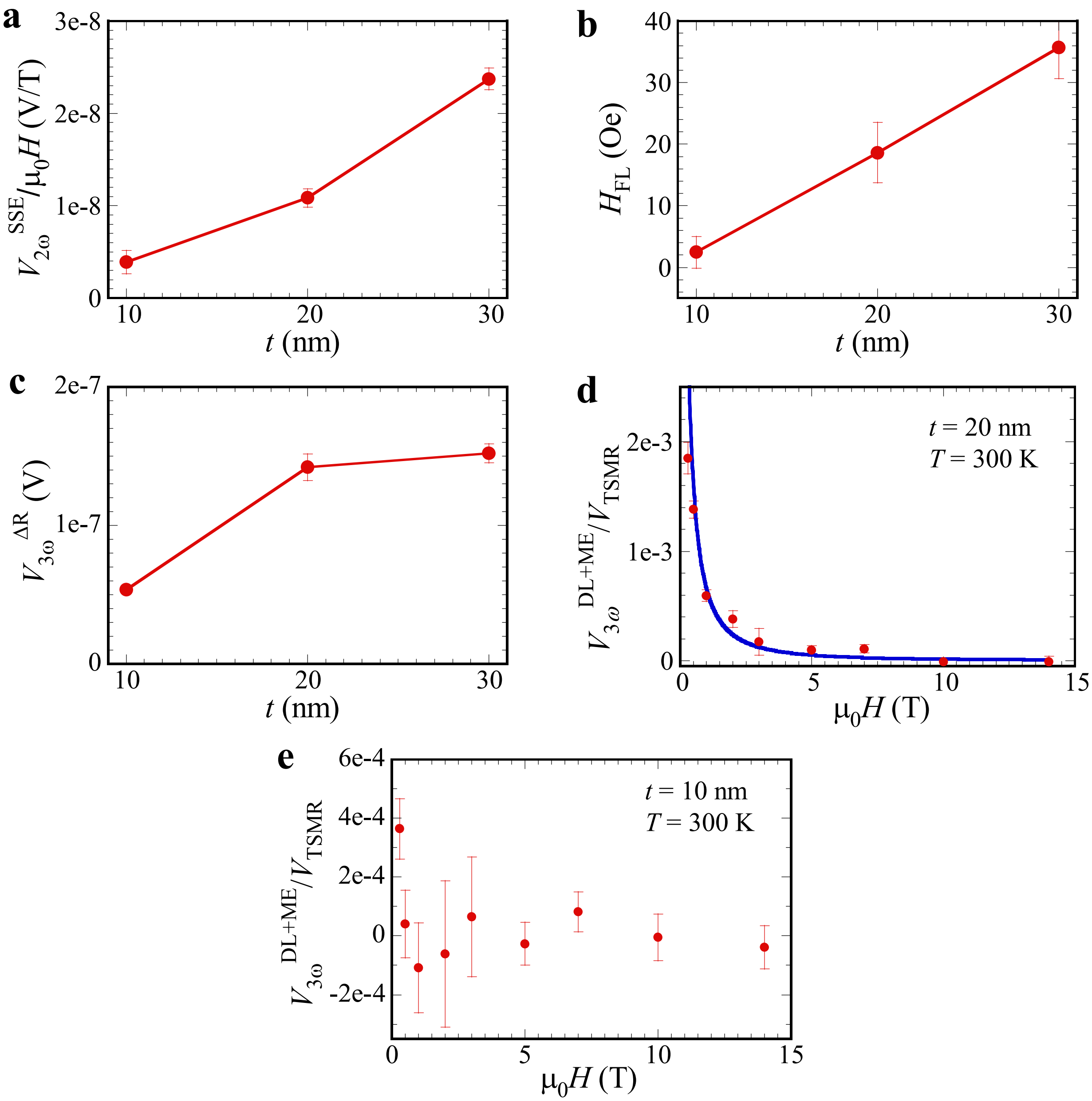

Figure S6. **Harmonic voltage components in Pt(5 nm)/*α*-Fe_2O_3 bilayers with different *α*-Fe_2O_3 thicknesses measured at 300 K**. **a**, Spin Seebeck effect coefficient. **b**, Effective field of field-like torque H_{FL}. **c**, Third harmonic voltage due to resistance change $V_{3\omega}^{\Delta \mathrm{R}}$. Normalized $V_{3\omega}^{\mathrm{DL+ME}}$ by V_{TSMR} as a function of applied magnetic field with **d**, 20 nm *α*-Fe_2O_3 and **e**, 10 nm *α*-Fe_2O_3. Error bars represent fitting uncertainty.

Figure S7 shows the different harmonic voltage components as a function of current. Based on the model we build using Eqs. S11-S18, it is expected that:

1) $V_{2\omega}^{\mathrm{SSE}}/H \propto I^2$

2) Extracted $H_{\mathrm{FL}} \propto I$

3) $V_{3\omega}^{\Delta\mathrm{R}} \propto I^3$

4) Extracted $H_{\mathrm{ME}} - H_{\mathrm{DL}}^{\mathrm{eff}} \propto I^2$.

These five components are plotted as a function of current in Figs. S7a-S7d and fitted by the corresponding polynomial functions with the expected current dependence.

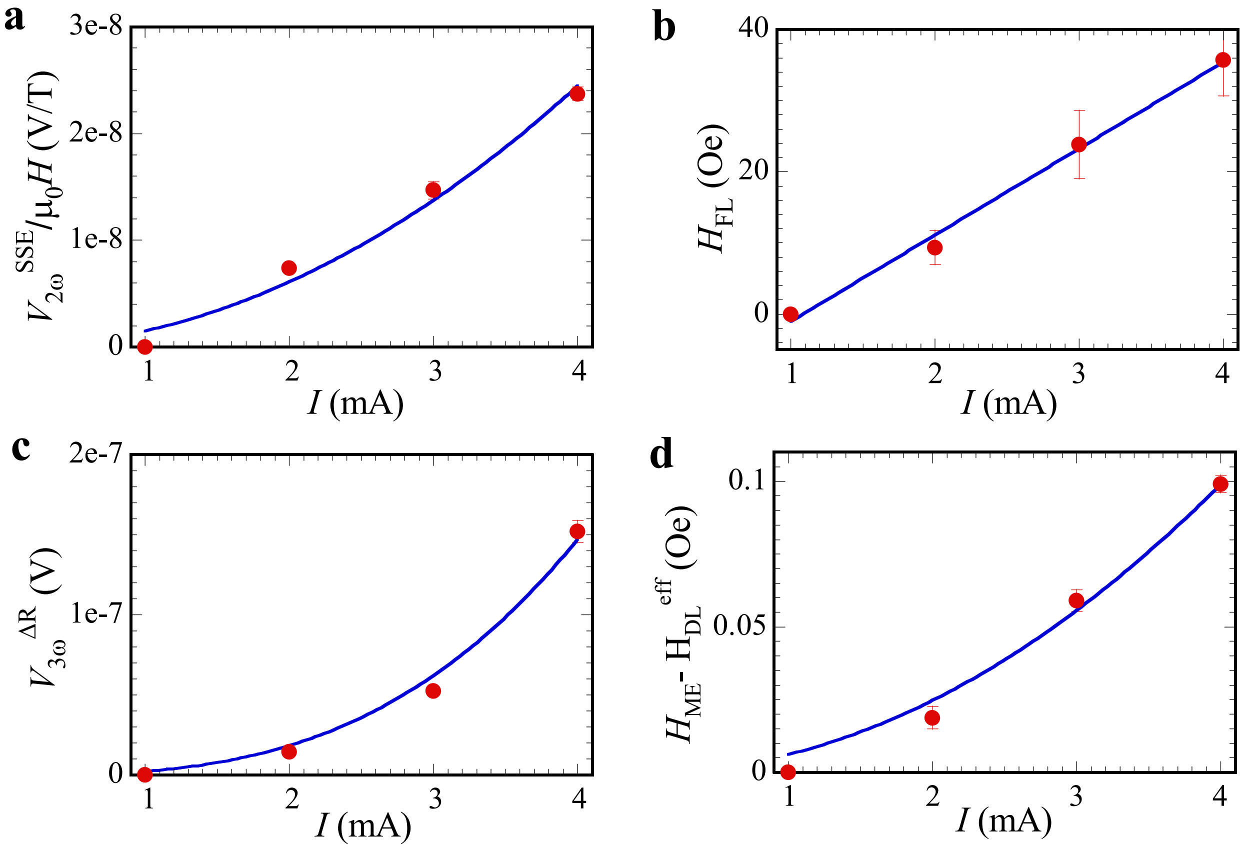

Figure S7. Harmonic voltage components in a Pt(5 nm)/α-Fe_2O_3 bilayer as a function of applied current measured at 300 K. a, Spin Seebeck effect coefficient. **b**, Effective field of field-like torque H_{FL}. **c**, Third harmonic voltage due to the resistance change $V_{3\omega}^{\Delta\mathrm{R}}$. **d**, Extracted $H_{\mathrm{ME}} - H_{\mathrm{DL}}^{\mathrm{eff}}$. Error bars represent fitting uncertainty.

5) Large imaginary part of spin mixing conductance in HM/magnetic-insulator heterostructures

The spin-orbit torque can be expressed as,[4]

$$\tau_{\text{SOT}} = \tau_{\text{DL-SOT}} + \tau_{\text{FL-SOT}} = \frac{\hbar}{2e}[G_r \mathbf{m} \times (\mathbf{m} \times \boldsymbol{\sigma})] + \frac{\hbar}{2e}[G_i \mathbf{m} \times \boldsymbol{\sigma}] \quad \text{(S19)}$$

Here, $G_{r(i)}$ is the real (imaginary) part of spin mixing conductance which determines the magnitude of DL(FL)-SOT. In HM/metallic-ferromagnet systems, $G_r \gg G_i$, which leads to a much larger H_{DL} compared with H_{FL}. This is confirmed by both experiments and first-principle calculations.[30,31] However, in HM/magnetic-insulator systems which receive much attention recently, this is not always the case. In Pt/$Y_3Fe_5O_{12}$ (YIG) and Pt/EuS bilayers,[32-34] much larger G_i than G_r has been reported. Large H_{FL} in our Pt/α-Fe_2O_3 might also be related to the insulating property of α-Fe_2O_3. To date, however, there are only few works that calculated the spin mixing conductance in HM/magnetic-insulator bilayers,[35] but failed to match the recent experimental results shown above. Further research in HM/magnetic-insulator systems is required address this question.

6) Thermal effect on the magnetic parameters

α-Fe_2O_3 has a very high Néel temperature ~950 K. Thus, its magnetic parameters, for example the sublattice magnetization, barely depend on temperature in our measurement temperature range. This has been confirmed in our previous work.[8] where the net magnetization measured in SQUID shows no difference at 300 K and 200 K. For temperature dependence of SOT effective field, it has been demonstrated that in HM/FM systems such as Co/Pt multilayers,[36] the monotonic increase of SOT effective field with temperature is mainly due to the increase of spin Hall efficiency of Pt instead of the decrease of saturation magnetization M_s. On the other hand, although current-induced temperature change is minor (<5 K) based on our COSMOL simulation, we still need to discuss its influence on our harmonic measurement. Since $R_{\text{TSMR}} \propto n_x n_y$, and the current-induced heating reduces n by $n(T) = n_0(1 - \alpha_n I^2)$,[37] $\Delta R_{\text{TSMR}} \propto 2\alpha_n n_{0x} n_{0y} I^2$ when ignoring higher order terms. Then we have $\Delta V = I \Delta R_{\text{TSMR}} \propto I^3$ with angular dependence $\sin 2\varphi_H$. This contribution can be merged into $V_{3\omega}^{\Delta R} = \frac{1}{8} \Delta V_{\text{TSMR}} \sin 2\varphi_H$. However, as mentioned above, the current induced temperature change is minor. Thus, we believe $V_{3\omega}^{\Delta R}$ is mainly originated from the current induced resistivity change of Pt.

References:

1 Zhang, P. X., Finley, J., Safi, T. & Liu, L. Q. Quantitative Study on Current-Induced Effect in an Antiferromagnet Insulator/Pt Bilayer Film. *Phys. Rev. Lett.* **2019**, 123, 247206.

2 Williamson, S. J. & Foner, S. Antiferromagnetic Resonance in Systems with Dzyaloshinsky-Moriya Coupling; Orientation Dependence in αFe_2O_3. *Phys. Rev.* **1964**, 136, A1102-A1106.

3 Fischer, J., Gomonay, O., Schlitz, R., Ganzhorn, K., Vlietstra, N., Althammer, M., Huebl, H., Opel, M., Gross, R., Goennenwein, S. T. B. & Geprägs, S. Spin Hall magnetoresistance in antiferromagnet/heavy-metal heterostructures. *Phys. Rev. B* **2018**, 97, 014417.

4 Chen, Y. T., Takahashi, S., Nakayama, H., Althammer, M., Goennenwein, S. T. B., Saitoh, E. & Bauer, G. E. W. Theory of spin Hall magnetoresistance. *Phys. Rev. B* **2013**, 87, 144411.

5 Seki, S., Ideue, T., Kubota, M., Kozuka, Y., Takagi, R., Nakamura, M., Kaneko, Y., Kawasaki, M. & Tokura, Y. Thermal Generation of Spin Current in an Antiferromagnet. *Phys. Rev. Lett.* **2015**, 115, 266601.

6 Bauer, G. E. W., Saitoh, E. & van Wees, B. J. Spin caloritronics. *Nat. Mater.* **2012**, 11, 391.

7 Avci, C. O. *Current-induced effects in ferromagnetic heterostructures due to spin-orbit coupling* Doctoral thesis, ETH Zürich, (2015).

8 Cheng, Y., Yu, S. S., Zhu, M. L., Hwang, J. & Yang, F. Y. Electrical Switching of Tristate Antiferromagnetic Néel Order in α-Fe_2O_3 Epitaxial Films. *Phys. Rev. Lett.* **2020**, 124, 027202.

9 Chiang, C. C., Huang, S. Y., Qu, D., Wu, P. H. & Chien, C. L. Absence of Evidence of Electrical Switching of the Antiferromagnetic Neel Vector. *Phys. Rev. Lett.* **2019**, 123, 227203.

10 Churikova, A., Bono, D., Neltner, B., Wittmann, A., Scipioni, L., Shepard, A., Newhouse-Illige, T., Greer, J. & Beach, G. S. D. Non-magnetic origin of spin Hall magnetoresistance-like signals in Pt films and epitaxial NiO/Pt bilayers. *Appl. Phys. Lett.* **2020**, 116, 022410.

11 Baldrati, L., Gomonay, O., Ross, A., Filianina, M., Lebrun, R., Ramos, R., Leveille, C., Fuhrmann, F., Forrest, T. R., Maccherozzi, F., Valencia, S., Kronast, F., Saitoh, E., Sinova, J. & Kläui, M. Mechanism of Neel Order Switching in Antiferromagnetic Thin Films Revealed by Magnetotransport and Direct Imaging. *Phys. Rev. Lett.* **2019**, 123, 177201.

12 Akiyama, T., Ohta, H., Takahashi, R., Waseda, Y. & Yagi, J.-i. Measurement and Modeling of Thermal Conductivity for Dense Iron Oxide and Porous Iron Ore Agglomerates in Stepwise Reduction. *ISIJ International* **1992**, 32, 829-837.

13 Saeki, I., Ohno, T., Seto, D., Sakai, O., Sugiyama, Y., Sato, T., Yamauchi, A., Kurokawa, K., Takeda, M. & Onishi, T. Measurement of Young's modulus of oxides at high temperature related to the oxidation study. *Materials at High Temperatures* **2011**, 28, 264-268.

14 Arblaster, J. W. Selected Values for the Densities and Molar Volumes of the Liquid Platinum Group Metals and of the Initial Melting Curves of Iridium, Rhodium and Ruthenium. *Johnson Matthey Technology Review* **2017**, 61, 80.

15 Zhang, X., Xie, H. Q., Fujii, M., Ago, H., Takahashi, K., Ikuta, T., Abe, H. & Shimizu, T. Thermal and electrical conductivity of a suspended platinum nanofilm. *Appl. Phys. Lett.* **2005**, 86, 171912.

16 Farraro, R. & McLellan, R. B. Temperature dependence of the Young's modulus and shear modulus of pure nickel, platinum, and molybdenum. *Metallurgical Transactions A* **1977**, 8, 1563-1565.

17 Wachtman, J. B., Tefft, W. E., Lam, D. G. & Apstein, C. S. Exponential Temperature Dependence of Young's Modulus for Several Oxides. *Phys. Rev.* **1961**, 122, 1754-1759.

18 de Faoite, D., Browne, D. J., Chang-Díaz, F. R. & Stanton, K. T. A review of the processing, composition, and temperature-dependent mechanical and thermal properties of dielectric technical ceramics. *J. Mater. Sci.* **2012**, 47, 4211-4235.

19 KÖSter, W. & Franz, H. Poisson's Ratio for Metals and Alloys. *Metallurgical Reviews* **1961**, 6, 1-56.

20 Furukawa, G. T., Reilly, M. L. & Gallagher, J. S. Critical Analysis of Heat—Capacity Data and Evaluation of Thermodynamic Properties of Ruthenium, Rhodium, Palladium, Iridium, and Platinum from 0 to 300K. A Survey of the Literature Data on Osmium. *J. Phys. Chem. Ref. Data* **1974**, 3, 163-209.

21 Snow, C. L., Shi, Q., Boerio-Goates, J. & Woodfield, B. F. Heat capacity, third-law entropy, and low-temperature physical behavior of bulk hematite (α-Fe2O3). *J. Chem. Thermodyn.* **2010**, 42, 1136-1141.

22 Powell, R. L. & Blanpied, W. A. *Thermal Conductivity of Metals and Alloys at Low Temperatures: A Review of the Literature*. (U.S. Government Printing Office, 1954).

23 Touloukian, Y. S. *Thermophysical properties of matter; [the TPRC data series; a comprehensive compilation of data]*. (IFI/Plenum, 1970).

24 Yates, B., Cooper, R. F. & Pojur, A. F. Thermal expansion at elevated temperatures. II. Aluminium oxide: experimental data between 100 and 800 K and their analysis. *J. Phys. C: Solid State Phys.* **1972**, 5, 1046-1059.

25 Kirby, R. K. Platinum—A thermal expansion reference material. *Int. J. Thermophys.* **1991**, 12, 679-685.

26 Dorogokupets, P., Ponomarev, E. M. & Melekhova, E. Optimization of experimental data on the heat capacity, volume, and bulk moduli of minerals. *Petrology* **1999**, 7, 574-591.

27 Wen, Y., Wu, J., Li, P., Zhang, Q., Zhao, Y. L., Manchon, A., Xiao, J. Q. & Zhang, X. X. Temperature dependence of spin-orbit torques in Cu-Au alloys. *Phys. Rev. B* **2017**, 95, 104403.

28 Qiu, X. P., Deorani, P., Narayanapillai, K., Lee, K.-S., Lee, K.-J., Lee, H.-W. & Yang, H. Angular and temperature dependence of current induced spin-orbit effective fields in Ta/CoFeB/MgO nanowires. *Sci Rep* **2014**, 4, 4491.

29 Kim, J., Sinha, J., Mitani, S., Hayashi, M., Takahashi, S., Maekawa, S., Yamanouchi, M. & Ohno, H. Anomalous temperature dependence of current-induced torques in CoFeB/MgO heterostructures with Ta-based underlayers. *Phys. Rev. B* **2014**, 89, 174424.

30 Avci, C. O., Garello, K., Gabureac, M., Ghosh, A., Fuhrer, A., Alvarado, S. F. & Gambardella, P. Interplay of spin-orbit torque and thermoelectric effects in ferromagnet/normal-metal bilayers. *Phys. Rev. B* **2014**, 90, 224427.

31 Zhang, Q. F., Hikino, S. & Yunoki, S. First-principles study of the spin-mixing conductance in Pt/$Ni_{81}Fe_{19}$ junctions. *Appl. Phys. Lett.* **2011**, 99, 172105.

32 Sun, Y. Y., Chang, H. C., Kabatek, M., Song, Y.-Y., Wang, Z. H., Jantz, M., Schneider, W., Wu, M. Z., Montoya, E., Kardasz, B., Heinrich, B., Velthuis, S. G. E. t., Schultheiss, H. & Hoffmann, A. Damping in Yttrium Iron Garnet Nanoscale Films Capped by Platinum. *Phys. Rev. Lett.* **2013**, 111, 106601.

33 Gomez-Perez, J. M., Zhang, X.-P., Calavalle, F., Ilyn, M., González-Orellana, C., Gobbi, M., Rogero, C., Chuvilin, A., Golovach, V. N., Hueso, L. E., Bergeret, F. S. & Casanova, F. Strong Interfacial Exchange Field in a Heavy Metal/Ferromagnetic Insulator System Determined by Spin Hall Magnetoresistance. *Nano Letters* **2020**, 20, 6815-6823.

34 Roy, K. Determining complex spin mixing conductance and spin diffusion length from spin pumping experiments in magnetic insulator/heavy metal bilayers. *Appl. Phys. Lett.* **2020**, 117, 022404.

35 Jia, X. T., Liu, K., Xia, K. & Bauer, G. E. W. Spin transfer torque on magnetic insulators. *Epl-Europhys Lett* **2011**, 96, 17005.

36 Chen, S. W., Li, D., Cui, B. S., Xi, L., Si, M. S., Yang, D. Z. & Xue, D. S. Temperature dependence of spin–orbit torques in Pt/Co/Pt multilayers. *J. Phys. D: Appl. Phys.* **2018**, 51, 095001.

37 Garello, K., Miron, I. M., Avci, C. O., Freimuth, F., Mokrousov, Y., Blügel, S., Auffret, S., Boulle, O., Gaudin, G. & Gambardella, P. Symmetry and magnitude of spin–orbit torques in ferromagnetic heterostructures. *Nat. Nanotechnol.* **2013**, 8, 587-593.
